# Role and Involvement of TENM4 and miR-708 in Breast Cancer Development and Therapy

**DOI:** 10.3390/cells11010172

**Published:** 2022-01-05

**Authors:** Giulia Peppino, Federica Riccardo, Maddalena Arigoni, Elisabetta Bolli, Giuseppina Barutello, Federica Cavallo, Elena Quaglino

**Affiliations:** Molecular Biotechnology Center, Department of Molecular Biotechnology and Health Sciences, University of Torino, Via Nizza 52, 10126 Torino, Italy; giulia.peppino@unito.it (G.P.); federica.riccardo@unito.it (F.R.); maddalena.arigoni@unito.it (M.A.); elisabetta.bolli@unito.it (E.B.); giuseppina.barutello@unito.it (G.B.); federica.cavallo@unito.it (F.C.)

**Keywords:** Teneurin 4 (TENM4), miR-708, breast cancer, tumor progression, metastasis, target, biomarker

## Abstract

Teneurin 4 (TENM4) is a transmembrane protein that is codified by the *ODZ4* gene and is involved in nervous system development, neurite outgrowth, and neuronal differentiation. In line with its involvement in the nervous system, TENM4 has also been implicated in several mental disorders such as bipolar disorder, schizophrenia, and autism. TENM4 mutations and rearrangements have recently been identified in a number of tumors. This, combined with impaired expression in tumors, suggests that it may potentially be involved in tumorigenesis. Most of the TENM4 mutations that are observed in tumors occur in breast cancer, in which TENM4 plays a role in cells’ migration and stemness. However, the functional role that TENM4 plays in breast cancer still needs to be better evaluated, and further studies are required to better understand the involvement of TENM4 in breast cancer progression. Herein, we review the currently available data for TENM4′s role in breast cancer and propose its use as both a novel target with which to ameliorate patient prognosis and as a potential biomarker. Moreover, we also report data on the tumorigenic role of miR-708 deregulation and the possible use of this miRNA as a novel therapeutic molecule, as miR-708 is spliced out from *TENM4* mRNA.

## 1. Introduction: Teneurin 4 (TENM4) Identification Card: Gene, Protein Structure and Functions

TENM4 is a transmembrane protein that is codified in chromosome 11 by the *ODZ4* gene, which includes 34 exons. While the CAAT enhancer binding protein Homologous Protein (CHOP) and Glucocorticoid Receptor-α (GRα) are experimentally established TENM4 activators, the C-Terminal Binding Protein 2 (CTBP2) is predicted to repress its expression [1]. Additional predicted proteins that bind to the *ODZ4* promoter include Myc and E2F Transcription Factor 1 (E2F1), though their role in regulating TENM4 expression has not yet been validated. Moreover, there are evidences to suggest that TENM4 expression in the nervous system is regulated by Empty Spiracles Homeobox 2 (EMX2) and Paired Box 6 (PAX6) proteins [2].

**Figure 1:**
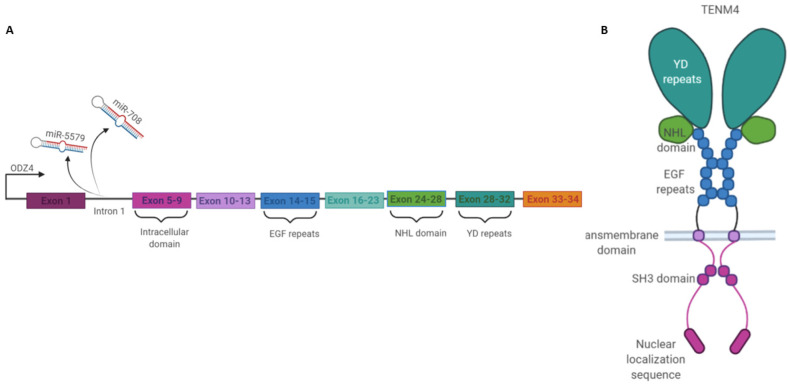
Schematic representation of the *ODZ4* gene and of the TENM4 protein structure: (**A**) The *ODZ4* gene counts 34 exons (NCBI Gene ID: 26011, updated on 23 November 2021). Two microRNA miR-5579 and miR-708 are situated in the first intron of the *ODZ4* gene and spliced out. (**B**) TENM4 protein structure comprehend different domains: an intracellular domain with a nuclear localization sequence and an SH3 binding domain, a transmembrane domain, and an extracellular domain composed by eight EGF repeats, an NHL, and YD domains. “Created with BioRender.com, accessed on 26 November 2021”.

Two microRNAs, named miR-5579 and miR-708, are situated in the first intron of the *ODZ4* gene (Figure 1A). While miR-5579 has been identified as being one of the overexpressed miRNAs in gastric cancer [3], little is known about its target and function in cancer cells. By contrast, more information is available on the contribution of miR-708 to carcinogenesis. MiR-708 is spliced out from *TENM4* mRNA, processed, and exported into the cytoplasm. Its loading into the RNA-Induced Silencing Complex (RISC) suggests that this miRNA is functional [4]. Moreover, the two *ODZ4* inducers, CHOP and GRα, are also able to activate miR-708 expression [5]. However, it should be noted that TENM4 and miR-708 expression are not always simultaneous in all tissues. While they are both highly expressed in the brain and eyes, in other tissues, such as in muscles, their expression is different, with miR-708 being more highly expressed than TENM4 [1,4]. The presence of different miR-708 enhancers in the *ODZ4* gene may be responsible for the different levels of TENM4 and miR-708 expression in tissues. Nevertheless, post-transcriptional processes should also be taken into consideration [1,6]. However, miR-708 regulation is still not fully understood.

TENM4 is a glycosylated type II transmembrane protein that belongs to the family of Teneurins (TENMs), which include four TENM members (TENM1–4). All TENMs show great organization homology, with a highly phylogenetically conserved C-terminal extracellular domain, a single-pass transmembrane sequence, and a variable small N-terminal intracellular domain. The TENM4 extracellular globular structure, as with those of the other proteins in the family, is made up of several binding motifs, including Epidermal Growth Factor (EGF)-like repeats that can mediate TENM4 *cis*-dimerization, followed by a beta propeller region with an ‘NCL-1, HT2A and Lin-41′ (NHL) domain that is required for homophilic TENM4 *trans*-interactions and a tyrosine–aspartate (YD) repeat region that contains a binding site that is needed for heterophilic protein communication (Figure 1B) [7,8,9,10,11,12]. As with the other TENMs, TENM4 can mediate cell–cell interactions with Latrophilins (LPHNs), which are a family of G-Protein Coupled Receptors (GPCR), and with the actin-crosslinking protein filamin [13]. X-ray crystallographic studies have demonstrated that the Fibronectin Leucine-rich Repeat Transmembrane protein (FLRT) is involved in TENM4–LPHN binding [14].

At the extremity of the C-terminal part of TENM4, as for all other TENMs proteins, lies a bioactive peptide named “Teneurin C-terminal Associated Peptide” (TCAP), which is 41 amino acids in length [15]. While TCAP-1 is also independently transcribed via an alternative promoter site and released [16], no such evidence has arisen for TCAP-4. Nevertheless, the presence of a convertase-like cleavage site at the amino terminus suggests that TCAP-4 could also be released from the rest of the protein [17,18]. However, few data are available for the role and mechanisms of action of TCAP-4, and deeper investigations are warranted.

While the extracellular domains of the different TENMs are highly homologous with only few functional differences, the intracellular sequence is less conserved between isoforms, and typically includes one or more proline-rich, SH3-binding domains [9,19]. TENM4, as with other TENMs, shows a cleavage site in the intracellular domain that allows the intracellular portion to be processed. Moreover, a predicted nuclear localization sequence has suggested that the TENM4 intracellular domain, as with those of TENM1 and TENM3, can be cleaved and translocated into the nucleus, where they can modulate gene expression [20].

All four TENMs have been implicated in the development of the nervous system [21]. Specifically, the expression of TENM4 is tightly regulated in space and time within the evolving nervous system and in the regulatory sites of morphogenesis. Several studies have demonstrated the presence of *TENM4* mRNA expression in the hippocampus, in the trigeminal ganglia, and in the cerebral cortex of developing mouse brains [22]. Moreover, considerable amounts of data have indicated the involvement of TENM4 in neuronal cell differentiation, as it is expressed by cortical neurons and their precursors in a high-caudal-to-low-rostral gradient, regulating cortical area patterning [2]. On differentiated neurons, TENM4 plays a key role in promoting neurite outgrowth via Focal Adhesion Kinase (FAK) signaling. Furthermore, TENM4 has been implicated in synapse formation, axon guidance, synapse specificity, and neuronal migration. It has also been suggested that TENM4 plays a role in nervous system development as a regulator of cell differentiation in oligodendrocytes [23]. As far as the function of TENM4 in the central nervous tissue is concerned, studies using TENM4 Knock-Out (KO) mice have demonstrated that TENM4 plays a role in the tremor phenotype. Indeed, TENM4 disruption impairs the normal oligodendrocyte differentiation process, leading to lower axon myelination, which is responsible for the essential tremor [23]. A similar phenotype has been identified in humans carrying a pathogenic missense mutation in TENM4 that leads to a threonine-to-asparagine amino acid substitution [24,25,26]. Although the relevance of this mutation was not confirmed when a Canadian population was analyzed [27], these results deserve further investigation to evaluate the potential association between TENM4 dysfunction, oligodendrocyte maturation and central myelination, and the genetic architecture of the essential tremor in humans. In line with the important role of TENM4 in central nervous system development, *ODZ4* has been identified as a risk gene for many types of mental diseases, including bipolar disorder (Psychiatric GWAS Consortium Bipolar Disorder Working Group, 2011) [28,29,30], schizophrenia [31,32], and autism [33].

There are few data on the function of TENM4, and, in general, of TENMs outside the nervous system, and the question remains quite unclear. TENM4 has been implicated in the differentiation of chondrocyte and satellite cells, the latter of which are also known as muscle stem cells [23,34,35,36]. In particular, TENM4 has been described as a suppressor of chondrogenic and myogenic differentiation in these cells, suggesting that TENM4 may play a dual role in cell differentiation, depending on the tissue and where it has its function. TENM4 is also expressed in non-neuronal developing tissues of mesodermal derivation, such as the mesentery, forming limb and bone, but also in the trachea, adipose tissue, and skin [37]. In developing mouse limbs, the depletion of the Homeobox D (HoxD) transcription factor, which is involved in morphogenesis, is associated with increased TENM4 expression, suggesting that TENM4 is a possible HoxD target [38]. In adult mice, low *TENM4* mRNA expression has also been observed in the liver, testes, and thymus, besides the brain [37,39]. Moreover, TENM4 variants have also been identified as potential genetic risks for type 2 diabetes mellitus [40] (GWAS and other genetic association datasets from the GWASdb SNP-Disease Associations dataset).

More recently, TENM4 has also been implicated in tumorigenesis [41,42]. However, its expression and function seem to be contradictory, with possible different tissue-specific regulation mechanisms and tumor-specific functions [43]. Herein, we review the currently available data for the role of TENM4 in breast cancer and propose it as a potential biomarker and novel target. Moreover, as miR-708 is spliced out from *TENM4* mRNA, we also report data on the tumorigenic role of miR-708 deregulation in different breast cancer subtypes. A deep knowledge of the role of both the TENM4 protein and miR-708 in breast cancer cells and tumors will facilitate the investigation of combinatorial strategies that aim to halt tumor progression and prolong cancer patient survival.

## 2. TENM4 and Cancer

TENM4 expression has been found to be deregulated in several tumor types. However, the data available on the role of TENM4 as an oncogene or oncosuppressor are limited and contradictory. Our recently published data, which were obtained using an in silico analysis of RNA sequencing data and the web-based tool Gene Expression Profiling Interactive Analysis (GEPIA; http://gepia.cancer-pku.cn, accessed on 28 January 2021), demonstrated that there is a significant increase in *TENM4* mRNA expression in human lung adenocarcinoma, lower grade glioma, and pancreatic adenocarcinoma compared to normal tissues. However, lower *TENM4* mRNA levels have been observed in skin cutaneous melanoma, ovarian serous cystadenocarcinoma, and in testicular germ cell tumors than in normal tissues [44]. Moreover, lower *TENM4* expression in human ovarian cancer has been more highly associated with undifferentiated tissues than differentiated ones and with reduced ovarian cancer cells’ sensitivity to cisplatin, demonstrating that, in this type of tumor, lower *TENM4* expression correlates with worse patient prognosis and possibly with tumor chemoresistance [43]. The opposite correlation trend between *TENM4* expression and prognosis, however, is observed in liver, endometrial, stomach, and renal cancer, in which a higher *TENM4* expression correlates with reduced patient survival [42]. In addition, the significant increase of *TENM4* mRNA levels that were observed in tumors, compared to normal colon tissues, in a mouse model of colorectal cancer suggest that *TENM4* may have a role in colorectal cancer development and progression [45]. These data suggest that TENM4 may have different functions in different tumors.

Besides mRNA and protein overexpression, a number of different mutations and chromosomal rearrangements have been described in different tumor types for the *ODZ4* gene, just as for the other TENM genes [42]. In particular, *ODZ4* is one of the most mutated genes in primary lymphoma. The majority of these mutations occur in the extracellular domain and may have a functional role in tumorigenesis. Regarding *TENM* rearrangements, 75% occur in *ODZ4*, suggesting that the *ODZ4* gene is more susceptible to chromosome breakage than other *TENMs* genes [42]. Of the rearrangements identified so far, *ODZ4* rearranges with Deleted in Lung and Esophageal Cancer protein 1 (*DLEC1*) and Mitochondrial Carrier Homolog 2 (*MTCH2*) in small cell lung carcinoma, and with DNA Polymerase Delta subunit 3 (*POLD3*) and Glutamate Ionotropic Receptor Kainate Type Subunit 4 (*GRIK4*) in lung adenocarcinoma [42,46,47,48,49]. In esophageal carcinoma, however, *ODZ4* rearranges with Exostosin Glycosyltransferase 2 (*EXT2*), while *ODZ4* rearranges with Cell Adhesion Molecule 1 (*CADM1*) and Gram Domain containing 1b (*GRAMD1B*) in chronic lymphocytic leukemia [42,46,48,50]. Additionally, chromothripsis, a process that causes a huge number of chromosomal rearrangements in a single event, has been shown to involve the *ODZ4* gene in small cell lung cancer as well as in neuroblastoma [47,51]. Finally, *ODZ4* has also been identified as an integration site of the hepatitis B virus in hepatocellular carcinoma [52].

## 3. MiR-708 and Cancer

MiRNA are potential regulators of tumorigenesis and do so via post-transcriptional gene repression. In this setting, the deregulation of miR-708 expression has been described in several types of cancers. In particular, its downregulation in tumor, as compared to normal tissues, has been observed in prostate, gastric, breast and ovarian cancer, melanoma, osteosarcoma, Ewing sarcoma, hepatocellular and renal cell carcinoma, and in chronic lymphocytic leukemia. However, in lung, colorectal, and bladder cancer, a higher miR-708 expression has been found in tumor, rather than in normal, tissues [1,53]. Whether miR-708 acts as a tumor suppressor or oncogene may depend on the different miR-708 genes it targets in different tumors. For instance, in prostate cancer, miR-708 downregulation induces, on one hand, increased Karyopherin α4 (KPNA4) expression, increasing cancer cell metastatization; however, on the other hand, the reduced targeting of pro-tumorigenic proteins such as CD44, AKT serine/threonine kinase 2 (AKT2), and Neuronatin (NNAT) increases cells malignancy [54,55]. Similarly, reduced miR-708 *Notch1* targeting in gastric cancer promotes cell proliferation and metastatization [56]. In hepatocellular carcinoma, miR-708 is involved in cell dissemination through SMAD Family Member 3 (*SMAD3*) targeting [57]. However, the tumor suppressor role of miR-708 in renal cell carcinoma is mediated by reduced Survivin, Zinc finger E-box-binding homeobox 2 (*ZEB2*), and FLICE-like Inhibitory Protein (*FLIP*) targeting. Indeed, the increased expression of Survivin and FLIP, which are antiapoptotic proteins, promotes cancer cell survival, while the overexpression of ZEB2, which is a regulator of E-cadherin, promotes cell migration and dissemination [58,59]. In Ewing sarcoma, miR-708 downregulation contributes to tumor progression via the consequently increased expression of EYA Transcriptional Coactivator and Phosphatase 3 (EYA3), which is a transcriptional activator involved in cancer cell survival, invasion, and chemoresistance [60]. The involvement of miR-708 downregulation in chemoresistance has also been demonstrated via *Caspase-3* targeting in ovarian cancer [61,62]. In melanoma, increased cell proliferation and invasion has been associated with the lower miR-708 targeting of Lymphoid Enhancer-binding Factor-1 (*LEF1*), which is a member of the Wnt pathway [63]. In osteosarcoma, miR-708 downregulation promotes cell growth and invasion via the Upregulator of Cell Proliferation (URGCP)/NF-κB pathway [64]. A similar oncogenic role for miR-708 has been observed in chronic lymphocytic leukemia, where its reduced expression affects NF-κB activity via the kinase-β/IKBKB (*IKKβ*) targeting [6]. A different scenario is found in lung, colorectal, and bladder cancer, where miR-708 is more highly expressed than in normal tissues and acts as an oncogene. In particular, miR-708-mediated targeting of *p21*, a proapoptotic protein, in lung cancer decreases cell death and increases cell migration [65]. Conversely, increased cell proliferation and colorectal cancer cell invasion is mediated by the targeting of the Cyclin-Dependent Kinase inhibitor 2B (*CDKN2B*) by miR-708 [66], while, in bladder cancer the targeting of *Caspase-2* by miR-708 reduces cell apoptosis [67]. A summary of miR-708 target genes in different tumors is reported in Table 1.

## 4. TENM4 and Breast Cancer

As previously discussed, there are data derived from genome-wide approaches that suggest preferential chromosome breakage at the *ODZ4* locus, with the privileged conservation of the 5′-N-terminal TENM4 domains [42]. Interestingly, most of the *ODZ4* rearrangements are found in breast cancer [42]. While no data are currently available on TENM4 expression and role in normal breast tissues, the first potential link between breast cancer and *ODZ4* was detected in the human breast cancer cell line MDA-MB-175. These cells harbor a translocation that involves *ODZ4* and *neuregulin (NRG1)* genes, which together generate a *ODZ4*/*NRG1* fusion gene. The new protein that is generated, called γ-heregulin, is controlled by the *ODZ4* promoter, and this fusion protein promotes cancer cell proliferation by constitutively activating the HER3–HER2 complex via γ-heregulin-dependent autocrine stimulation [68]. Since this fusion protein is not expressed in healthy breast tissue, it was initially proposed as a potential oncogene that was involved in normal tissue transformation [68,69]. However, this translocation was not identified in other breast cancer cell lines, suggesting that γ-heregulin is not sufficiently widespread to be considered an oncogenic driver for breast cancer initiation [70]. The *ODZ4/NRG1* translocation is not the only rearrangement in which *ODZ4* is involved. Among the different genes involved in *ODZ4* rearrangements in breast cancer tissues, we can find RNA Binding Motif Protein 4 (*RBM4*), Transmembrane O-Mannosyltransferase Targeting Cadherins 2 (*TMTC2*), Adipogenesis Associated Mth938 Domain Containing (*AAMDC*), Anoctamin 10 (*ANO1*), chromosome 11 open reading frame 30 (*EMSY*), C2 Domain Containing 3 Centriole Elongation Regulator (*C2CD3*), DLEC1 cilia and flagella associated protein (*DLEC1*), GRB2 Associated Binding Protein 2 (*GAB2*), Kinesin Family Member 13B (*KIF13B*), Potassium Channel Tetramerization Domain Containing 21 (*KCTD21*), Mitochondrial Carrier 2 (*MTCH2*), Asparaginyl-TRNA Synthetase 2, Mitochondrial (*NARS2*), DNA Polymerase Delta 3, Accessory Subunit (*POLD3*), Prolyl Endopeptidase (*PREP*), Remodeling and Spacing Factor 1 (*RSF1*), and SH3 and Multiple Ankyrin Repeat Domains 2 (*SHANK2*) [42,46,48,49,71,72]. However, whether these rearrangements are functional and play a role in tumorigenesis has not yet been investigated.

**Figure 2:**
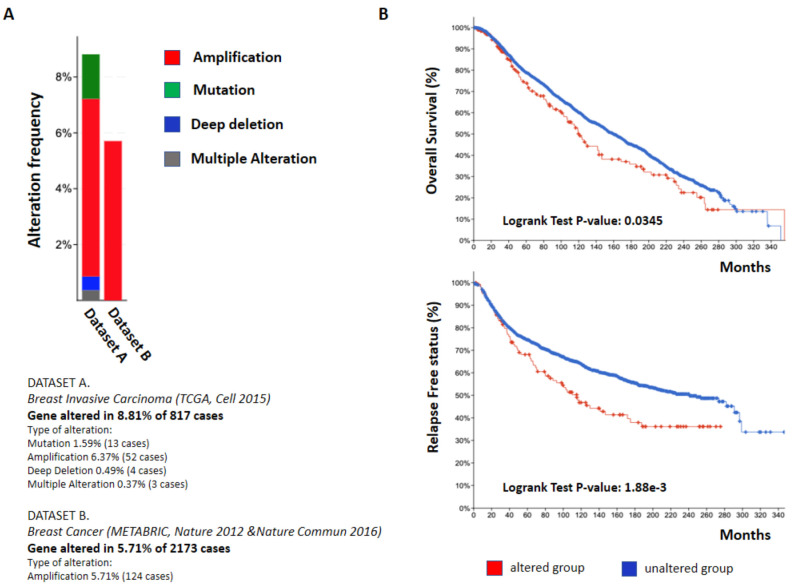
Analysis of *ODZ4* genomic alterations in breast cancer patients: (**A**) *ODZ4* alteration frequency observed by analyzing two breast cancer data sets: TCGA (Dataset A) and Metabric (Dataset B). The analyses were performed using the cBioPortal tool (www.cbioportal.org, accessed on 30 November 2021), an open-access, open-source resource for interactive exploration of multidimensional cancer genomics data sets. (**B**) Overall survival (upper panel) and relapse free status (lower panel) of breast cancer patients that displayed unaltered *ODZ4* (blue line) and genomic *ODZ4* alterations (red line). Statistical analysis was performed using the Log Rank Test.

Interestingly, an in silico analysis, which was performed by querying one of the most relevant published datasets on breast cancer patients, comprising 817 tumors, matched normal specimens [73] in order to explore the genetic alterations that might occur at the *ODZ4* gene level, revealed that the gene is altered in about 9% of cases. The amplification of the gene was detected in most cases (6.7%), while gene mutations have been found in about 1.5%, deep deletion in 0.5%, and multiple alterations in only 0.3% of the patients analyzed (Figure 2A). The frequencies of *ODZ4* gene amplifications in breast cancer patients have also been confirmed in the in silico analysis of a second dataset that compares primary tumors with normal, noncancerous samples and peripheral blood cells [74] (Figure 2A). A summary of TENM4 mutation in breast cancer is reported (Figure S1 and Table S1). Remarkably, from a clinical point of view, the presence of gene alterations in breast cancer patients, regardless of their type, is significantly associated to a worse prognosis (Figure 2B).

Moving beyond gene alterations, an in silico analysis, performed using several publicly available datasets, showed that there is a trend of increased *TENM4* mRNA expression in breast cancer as compared to normal tissues [44]. Moreover, high TENM4 protein expression has been detected in different human and mouse breast cancer cell lines, including Estrogen Receptor (ER), Human Epidermal growth factor-2 (HER2), and positive and Triple Negative Breast Cancer (TNBC) subtypes (BT474, MCF-7, MDA-MB-231, T47D, HCC-1806, 4T1, and ZR7S) [43,75]. However, very few data are available on the functional role of TENM4 in breast cancer. The ability of TENM4 to induce neurite outgrowth and filopodia formation through FAK signaling suggests that TENM4 may have a role to play in cell migration [35]. Indeed, TENM4 knockdown reduces the formation of filopodia and FAK and Cdc42 and Rac1 activation, while its overexpression promotes neurite outgrowth [35]. Current findings show that TENM4 silencing, using specific siRNA in murine and human TNBC cell lines, is associated with significantly decreased cells’ migration ability and FAK phosphorylation, suggesting that TENM4 contributes to TNBC progression and cell dissemination [75]. However, there is still a lack of in vivo studies on TENM4 function, thus limiting current knowledge on the role of TENM4 in tumorigenesis and the molecular mechanisms through which it contributes to carcinogenesis.

Few research lines have focused on the potential implication of TENM4 in cancer cell stemness. Cancer Stem Cells (CSC) are characterized by self-regeneration and differentiation abilities and are involved in tumor metastatization, recurrences, and resistance to therapies. For this reason, the identification of targets that are specifically expressed on CSC could facilitate their eradication and improve patient outcomes [76]. Interestingly, TENM4 overexpression has been detected in CSC-enriched tumorspheres that were derived from murine and human breast cancer cell lines, including 4T1 (murine TNBC), HCC-1806 (human TNBC), MDA-MB-231 (human TNBC), MCF-7 (human ER-positive) and SK-BR-3 (human HER2-positive). Moreover, TENM4 silencing through specific siRNA has been found to be associated with the significant impairment of tumorsphere generation and the decreased expression of CSC markers such as the percentage of CD44^+^/CD24^−^ cells, the Octamer-binding Transcription factor 4 (OCT4), CD49f, and Aldehyde Dehydrogenase (ALDH) activity [75]. All these data suggest that, in different breast cancer subtypes, TENM4 plays a role in, tumorsphere formation, and self-renewal, and that it contributes to the malignant phenotype of breast cancer cells, likely by promoting metastatic progression and possibly drug resistance. However, to the best of our knowledge, the involvement of TENM4 in chemotherapy resistance in breast cancer has not yet been thoroughly investigated.

## 5. MiR-708 and Breast Cancer

There are some data that demonstrate that miR-708 plays a role in breast cancer cells’ migration, proliferation, and chemoresistance. However, unlike TENM4, it acts as a tumor suppressor [77,78]. Indeed, miR-708 expression is lower in breast cancer than in normal breast tissue and is even more suppressed in the metastasis and lymph nodes of metastatic breast cancer patients [77]. Its downregulation in these patients seems to be caused by a trimethylation in the H3K27 histone that is induced by Polycomb Repressor Complex 2 (PRC2). MiR-708 repression results in an increase in the expression of its targets *Neuronatin*, whose increased expression caused by miR-708 downregulation, is capable of inducing a decrease in Ca^2+^ levels that consequently increases ERK and FAK activation, resulting in higher MDA-MB-231 cell migration [77]. *Neuronatin* is not the only target that may explain miR-708 involvement in breast cancer cell migration. Another miR-708 target is Lysine-Specific histone Demethylase 1 (*LSD1*), which is a histone demethylase that affects breast cancer cell growth and migration through gene expression inhibition. Its overexpression in tumor tissues, caused by miR-708 downregulation, increases cell proliferation and invasion, and thus contributes to breast cancer malignancy [79].

MiR-708 is also involved in the Epithelial to Mesenchymal Transition (EMT), which has an important role in breast cancer cell migration [80]. MiR-708 downregulation in different breast cancer cell lines causes an increase in the expression of three of its direct targets, Zinc Finger E-Box Binding Homeobox 1 (*ZEB1*), Cadherin 2 (*CDH2*), and *vimentin*, which are known EMT inducers [80]. This miR-708 downregulation-mediated effect reduces the expression of E-cadherin and induces β-catenin, which are a negative regulator and an activator of EMT, respectively [80].

MiR-708 is also involved in chemoresistance. Indeed, its inhibition in different breast cancer cell lines increases resistance to chemotherapy, while its overexpression enhances cell sensitivity to both doxorubicin and docetaxel treatments [78,80]. Moreover, the reduced expression of miR-708 was detected in MCF-7 human breast cancer cells that were more resistant to adriamycin compared to parental ones [78].

A function in breast cancer stemness has also been described for miR-708. Unlike observations for TENM4, miR-708 expression is lower in tumorspheres derived from MDA-MB-231 and MCF-7 epithelial cell lines than in epithelial cells, and an analysis of a CD44^+^/CD24^−^ population revealed its downregulation [78]. Moreover, its knockdown increases tumorsphere formation, while its overexpression reduces the population of CD44^+^/CD24^−^ cells. This effect is a consequence of miR-708′s ability to target Cluster of Differentiation 47 (*CD47*), which is a glycoprotein that inhibits macrophages’ phagocytosis [78]. CD47 expression is increased in breast CSC and protects them from phagocytosis, but it also regulates their self-renewal capacity [81]. MiR-708 downregulation in tumorspheres increases CD47 expression and consequently inhibits CSC phagocytosis [78]. Moreover, miR-708 also targets NF-κB-activating kinase β (*IKKβ*), a kinase involved in CSC self-renewal through the NF-κB pathway. Indeed, miR-708 downregulation increases IKKβ and NF-κB expression, increasing cell proliferation, CSC phenotype, tumor growth, and metastasis in mice injected with breast CSC [82]. A summary of miR-708 target genes in breast cancer is reported in Table 2.

## 6. TENM4 as a Marker and Possible Target for Breast Cancer Treatment

**Figure 3:**
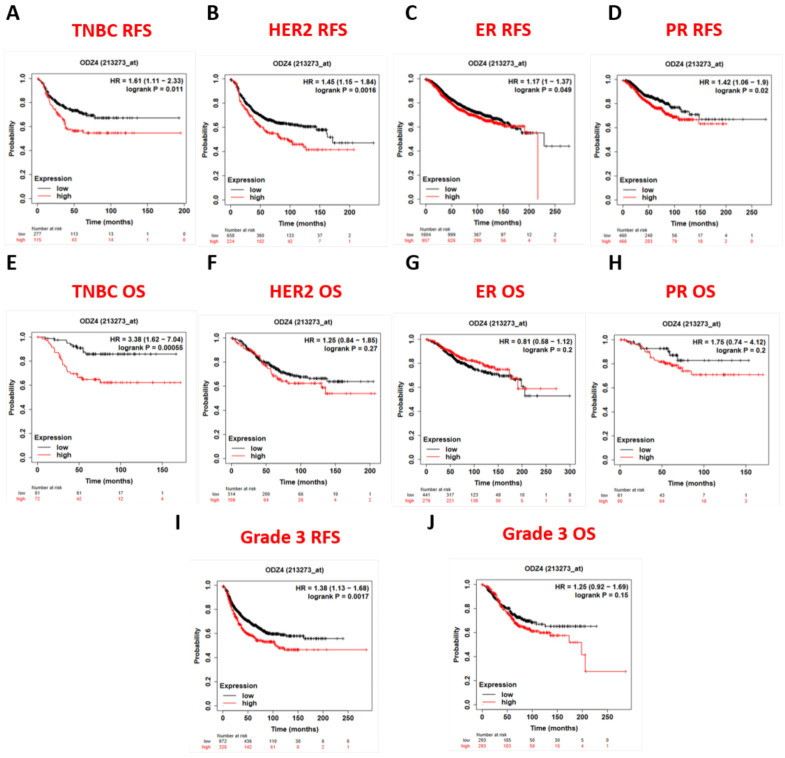
*TENM4* mRNA expression relevance in breast cancer patients. Correlation between Relapse Free Survival (RFS) and Overall Survival (OS) in Triple Negative Breast Cancer (TNBC) (**A**,**E**), HER2-positive (**B**,**F**), Progesterone Receptor (PR)-positive (**C**,**G**), Estrogen Receptor (ER)-positive (**D**,**H**), and Grade 3 (**I**,**J**) with high (red) or low (black) *TENM4* mRNA expression in the tumor.

No data on TENM4 targeting are currently available. However, its overexpression in different breast cancer cell subtypes other than in CSC and its involvement in promoting cancer cell migration [75] suggest that TENM4 may be clinically relevant for breast cancer. In order to investigate possible correlations between *TENM4* overexpression and patient prognosis, a meta-analysis using publicly available datasets of breast cancer patients was performed using Kaplan–Meier Plotter free software [83] (http://kmplot.com/analysis/index.php?p=service&cancer=breast, accessed on 17 May 2021). This in silico analysis shows that higher *TENM4* expression significantly correlates with worse patient Relapse-Free Survival (RFS) in all the breast cancer subtypes analyzed, including TNBC, HER2-positive, Progesterone Receptor (PR)-positive, and ER-positive breast cancers (Figure 3A–D). Interestingly, a significant correlation between *TENM4* overexpression and worse patient Overall Survival (OS) was found in TNBC. A trend can also be observed for HER2-positive and PR-positive breast cancer patients (Figure 3E–H). The possible involvement of *TENM4* overexpression and breast cancer aggressiveness is also indicated by its correlation with a worse RFS and OS in grade 3 breast cancer patients (Figure 3I,J), unlike in grade 1 and 2, where no correlation between *TENM4* expression and patient RFS and OS was observed (data not shown). These data indicate that it may be possible to use *TENM4* as a biomarker for patient survival, not only in TNBC, as we have previously shown [75], but also in patients bearing HER2- and PR-positive breast tumors. The limited yet increased RFS and the lack of significantly worse OS in ER-positive patients expressing high *TENM4* levels suggest that further studies are still required to understand whether *TENM4* could be used as a target in these patients.

According to these in silico results, TENM4 may be a promising target for cancer therapy in breast cancer patients that show TENM4 overexpression. Moreover, as TENM4 is a transmembrane protein whose expression is limited during development, and since low expression has been found in few adult tissues, it may be considered a good target for drug and immunotherapeutic approaches. Its expression in both differentiated epithelial cells and undifferentiated CSC means that its targeting may be beneficial for limiting tumor burden, but, most importantly, for eradicating CSC, thus limiting tumor cells dissemination. The development of vaccines coding for TENM4 would facilitate the generation of a long-lasting immune humoral and cellular response that could counteract breast tumor progression.

Our recently published data demonstrate that TENM4 can be detected in TNBC patient plasma and exosomes in higher amounts than in healthy donors [75]. Although further studies are still required to better understand the correlation between TENM4 expression in tissues and its release in sera. Our preliminary results suggest that there is also a potential use for TENM4 as a biomarker for breast cancer diagnosis, progression, and response to therapy. Moreover, since a proteomic study has reported the presence of TENM4-derived peptides in the urine of healthy donors [84], it would be interesting to analyze whether there are differences in TENM4 release in the urine of breast cancer patients compared to healthy donors. As the collection of urine is a noninvasive process for patients, and as urine is a better source for mass spectrometry than plasma and sera [84], TENM4 detection in urine would be a novel and more informative means with which to follow TENM4 release by breast cancer cells, thus contributing to establishing a new biomarker for breast cancer detection, prognosis, and response to therapy.

## 7. MiR-708 as a Marker and Possible Target for Breast Cancer Treatment

**Figure 4:**
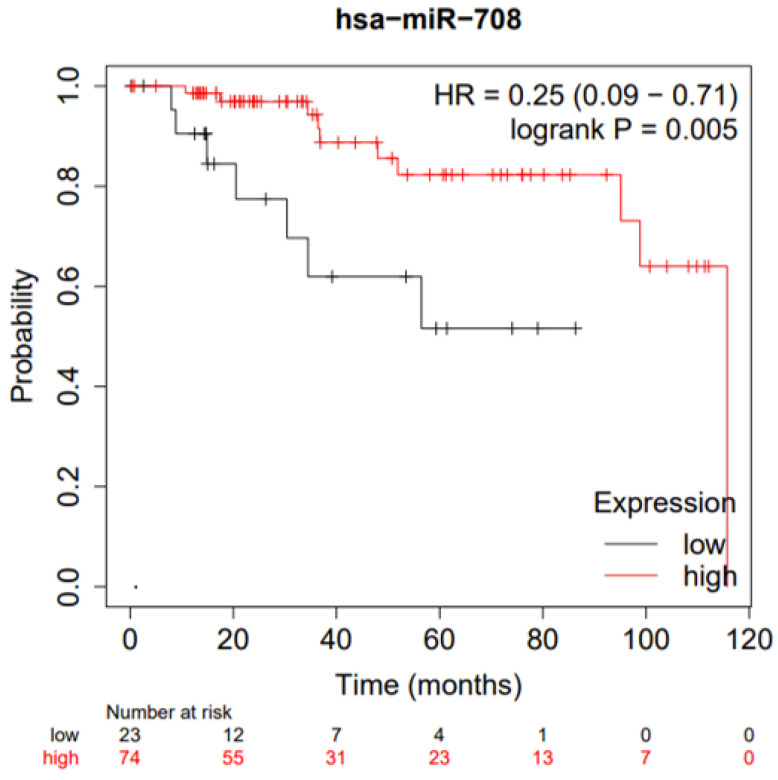
Mir-708 relevance in TNBC patients. Correlation between Overall Survival (OS) in TNBC and high (red) or low (black) miR-708 expression in the tumor.

The role of miR-708 in cancer cell migration and metastatization, as well as in chemoresistance, make it a good target for breast cancer patients [77,78]. Indeed, the in silico analysis of publicly available data sets show that TNBC patients with higher miR-708 expression have prolonged OS compared to those with lower expression (Figure 4). This is consistent with the role that miR-708 plays in tumor growth and metastatization [77,78] and with a recent publication demonstrating that a nanoparticle system, which is able to deliver miR-708 directly to the tumor in a TNBC mouse model is safe and effective in reducing the number of lung metastases in treated mice [85]. Furthermore, recent data has demonstrated that miR-708 expression is higher in patients that respond to chemotherapy than in non-responders [78]. All these reasons suggest that miR-708 may be a novel target for breast cancer treatment and an interesting predictive biomarker for therapy response in breast cancer patients.

While the interaction between TENM4 and miR-708 has not yet been studied, their opposite effects on cell migration and stemness may suggest that combined therapy might strengthen the antitumor effect. Indeed, higher TENM4 expression increases cancer cells stemness, whereas higher miR-708 expression decreases stemness. Similarly, higher TENM4 expression is required for higher tumor cell migration, while higher miR-708 expression inhibits their ability to migrate. Combined miR-708 delivery and TENM4 inhibition may potentially decrease cell migration and stemness and thus improve patient outcomes.

## 8. Do We Have Suitable Models for the Study of TENM4 in Breast Cancer Carcinogenesis?

Tumor cell lines are undoubtedly a relevant tool for the in vitro investigation of many aspects of cancer biology, such as genetic, epigenetic, and cellular pathway alterations [86,87]. In fact, breast cancer cell lines have, for many years, been the experimental model of choice for the in vitro investigation of how proliferation, migration, and apoptosis can be deregulated during disease progression [86,88]. Several well-characterized human breast cancer cell lines that share many of the genetic and genomic features of the most common clinical subtypes have been exploited for these purposes, and these include MCF-7 and T47D (luminal A), BT474 and MDA-MB-361 (luminal B), SKBR3 and HCC202 (HER2-positive), and BT20, MDA-MB-231, and MDA-MB-468 (TNBC). Nevertheless, there can be no doubt that in vitro investigations do not faithfully and comprehensively recapitulate what happens in vivo.

As a step forward for a more faithful representation of the human breast cancer in toto, innovative lines of research include looking towards the development of three-dimensional (3D) models called “organoids”. Many protocols have been developed and improved for the generation of breast organoids, with the inclusion of fresh tissue in different types of matrices and media components [89]. Breast organoids can be derived from normal breast tissue and are being genetically engineered with different technologies, including the CRISPR/Cas9 [90], allowing for the exploration of the relevance of tumor-suppressors or oncogenes, including TENM4, in breast cancer initiation and progression. Moreover, organoids can be derived from breast cancer biopsies, showing a high capability of reproducing the heterogeneity of cancer subtypes and of recapitulating some features of the in vivo breast microenvironment. Interestingly, breast cancer organoids have demonstrated a strong ability to retain the histopathology, hormone receptor status, and HER2 receptor status [91] of the original breast tumor, allowing a precise study of patient-specific cancer biology and drug response. Investigating TENM4 in established patient-derived breast cancer organoids could represent an unexplored, though appealing, tool with which to gain novel insights into the role of this oncogene, not only for breast cancer development but also for its treatment. However, it must be considered that organoid models are still limited in recapitulating the whole native breast tissue matrix composition and structure, as well as the in vivo tumor cell/stromal cell/matrix interactions.

As a consequence, different animal model systems have been developed with the intention of better simulating the natural evolution of breast cancer in patients and collecting new insights into the mechanisms that support the development of the disease, as well as evaluating the clinical potential of new therapies in vivo.

One of the most commonly used model systems exploits the engraftment of human breast cancer cell lines into immunocompromised animals. A great deal of attention has been dedicated to the characterization of the MDA-MB-231 cell line, which is commonly used to investigate the molecular basis of TNBC and the potential efficacy of experimental therapies against this type of breast cancer. Interestingly, explanted tumors derived from MDA-MB-231 cells that were subcutaneously injected into NOD/SCID-Gamma null (NSG) mice expressed high levels of TENM4, demonstrating that TENM4 protein expression is not restricted to cells that are cultured in vitro, but that it is retained during cancer growth in vivo [75]. Moreover, in this setting, TENM4 was shed by cancer cells in vivo, allowing the detection of an increase in circulating TENM4 in the plasma from MDA-MB-231-tumor-bearing mice compared to healthy NSG mice [75]. Overall, these results indicated the relevance of exploiting MDA-MB-231 cells in vivo as a model of TENM4-expressing TNBC.

However, although the use of xenografts with human breast cancer cell lines in NSG mice provide relevant information about the genetic and biological processes of tumors, as well as likely metastatic potential, accurately modeling the native associated microenvironment is difficult due to the need to use immunocompromised recipients, which lack an effective immune system. The use of murine breast cancer cell lines that can be injected into immunocompetent syngeneic mice is an appealing alternative means of achieving this goal, as it allows proper interactions to occur between the cancer and the host microenvironment cells, including those belonging to the immune system. However, few murine cell lines have been deeply characterized and spontaneously metastasize, reproducing the aggressive nature of human breast cancer. One of these includes the 4T1 cell line, which is archetypal of the human TNBC subtype [92,93,94]. Besides being detected in 4T1 cells cultured in 2D, interestingly, heterogeneous levels of TENM4 protein were also detected in 100% of established 4T1-tumors that were explanted from BALB/c mice, demonstrating that TENM4 protein expression is retained in vivo during cancer growth, even in this immunocompetent model [75]. In parallel to what was observed with MDA-MB-231, TENM4 release occurs in the plasma in this setting as well, with there being an increasing trend in circulating TENM4 in tumor-bearing 4T1 compared to healthy BALB/c mice [75].

Moving forward, these cell lines may also serve as models with which to investigate the role of TENM4 in tumor-initiating cells in vivo. Indeed, as described above, TENM4 upregulation has being demonstrated not only in epithelial cells, but also in both 4T1- and MDA-MB-231-derived tumorspheres enriched in CSC, suggesting that TENM4 may be involved in CSC self-renewal via the regulation of the FAK signaling pathway [75]. Overall, these results provide interesting groundwork for the use of MDA-MB-231 and 4T1 tumors for the investigation of the in vivo consequences of TENM4 targeting in the more staminal niche of TNBC tumors.

**Figure 5:**
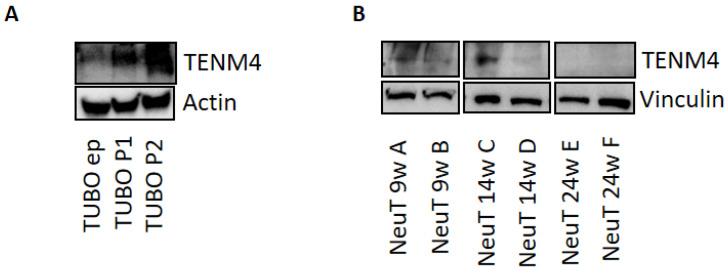
TENM4 expression in HER2-positive breast cancer cells and tumors: (**A**) Immunoblot of TENM4 and loading control protein (Actin) that compares HER2-positive TUBO epithelial cells (ep), first-passage tumorspheres (P1) and second-passage tumorspheres (P2). (**B**) Immunoblot of TENM4 and loading control protein (Vinculin) that compares HER2-positive mammary tumors from 9- (NeuT9wA and B), 14- (NeuT14wA and B), and 24- (NeuT24wA and B) week-old BALB-neuT mice. A total of 80 µg of TUBO cells and BALB-neuT tumor lysates were separated via electrophoresis in a 7.5% Mini-Protean TGX precast gel (Bio-Rad) and transferred onto an Immobilon-P PVDF membrane. Following blocking with 5% non-fat dry milk, the membrane was incubated with sheep anti-TENM4 (1 µg/mL, Cat#AF6320, R&D Systems, Minneapolis, MN, USA), with mouse anti-β-Actin (1:200, Clone AC-15, Santa Cruz Biotechnology), and with mouse anti-Vinculin (1:8000, produced in-house).

According to in silico data that show a correlation between TENM4 overexpression and shorter RFS and OS in HER2-positive breast cancer patients, and our previous findings that higher *TENM4* levels have been detected in a small cohort of HER2-positive breast cancer patients than in healthy donors [75], the use of preclinical models of HER2-positive breast cancer would be a helpful method with which to better investigate the role of TENM4 and the effects of in vivo TENM4 targeting in this subtype. Interestingly, TENM4 has been detected in a murine HER2-positive mammary cancer cell line, named TUBO (Figure 5A), meaning that there may be a new preclinical mouse model potentially available for the study of the role of TENM4 in the HER2-positive breast cancer subtype. Moreover, a significant increase in TENM4 expression has been found in TUBO-derived tumorspheres compared to epithelial cells (Figure 5A), making them an appealing cell line to be used in vivo to investigate the role of TENM4 in more staminal subsets of potential tumor-initiating cells in a context of HER2 activation.

Despite the relevance of mouse models that are based on cell-line-derived engrafts, we must consider that the breast cancer cells that are used are frequently derived from highly aggressive malignant tumors or plural effusions, making these models less suitable for modeling early events in the evolution of a primary tumor. Moreover, no single cell line is truly representative of the heterogeneity that is observed in primary breast cancers. In this landscape, Genetically Engineered Mice (GEM) come into their own, and provide an appealing model for the investigation of the multi-step process of breast cancer initiation and progression. BALB-neuT mice, which are transgenic for the rat HER2/neu oncogene, which is, itself, constitutively overexpressed under the transcriptional control of the Mouse Mammary Tumor Virus promoter Long Terminal Repeats (MMTV-LTR) promoter, is the most relevant GEM that has been exploited for the thorough investigation of the sequential steps of HER2-positive breast cancer [95]. All females develop mammary carcinomas with 100% penetrance, faithfully mimicking the natural evolution of metastatic HER2-positive breast cancer in human patients. BALB-neuT have been, and continue to be, used to investigate not only the biology of early and late stages of the disease, but also the effectiveness of experimental therapies and immunotherapies. Recent and preliminary data have provided evidence regarding TENM4 expression in BALB-neuT mammary tumors, with a higher level of expression in neoplastic lesions from 9- and 14-week-old animals and a significant decrease in the tumors from 24-week-old BALB-neuT mice (Figure 5B). These data will open up the possibility of investigating the role of TENM4 in the initiation and progression of HER2-positive breast cancer, in cancer cell dissemination, which, in this mouse model, is an early event in tumorigenesis and TENM4′s contribution to the metastatization process. Furthermore, the effectiveness of anti-TENM4 immune-targeting can be investigated in this spontaneous and well-characterized immunocompetent mouse model of HER2-positive breast cancer.

## 9. Conclusions

Breast cancer is currently treated based on the expression of the ER, PR, or HER2 receptors, and targeted therapies have led to considerable success in treating some breast cancer subtypes. Nevertheless, there is still no real effective targeted therapy for TNBC, which is one of the most aggressive subtypes of the disease. In addition, whilst primary breast cancer is highly treatable (80–99% of women diagnosed with stage I/II breast cancer survive to 5 years) (cancerresearchuk.org; wcrf.org, access date 30 November 2021), there is currently no effective cure available for metastatic breast cancer.

On the basis of these considerations, we have recently focused our attention on the identification of new potential targets in TNBC, and have identified TENM4 as a novel tumor-associated antigen in this subtype [44,75]. Interestingly, besides being expressed on murine and human epithelial TNBC tumor cells, TENM4 was found to be further upregulated in murine and human TNBC-CSC [44,75]. Subsequently, we demonstrated that TENM4 expression is not limited to TNBC cells, but that it is also found in cells from other breast cancer subtypes.

As discussed above, *ODZ4* was found to be altered, in terms of gene rearrangements, mutations, and overexpression, in all of the breast cancer subtypes analyzed, with there being a clinical correlation with worse patient RFS and OS in at least TNBC-, HER2-, and PR-positive patients. The possible involvement of *TENM4* overexpression in breast cancer aggressiveness has also been suggested by its correlation with a worse RFS and OS in grade 3 breast cancer patients. These results indicate that there may be a clinical role for *TENM4* as a new potential marker for prognostic and diagnostic purposes.

From a therapeutic point of view, TENM4 has the features of a good tumor-associated antigen that can be used for targeting via immunological approaches, including anti-cancer vaccines. Indeed, it has restricted expression in adult tissues, while it is highly expressed in breast cancer cells and has a relevant role in cancer progression, as it is overexpressed, not only by epithelial cells, where it can regulate migration and EMT, but it also seems to play a role in breast CSC [75]. On these bases, its immune targeting would result not only in the eradication of the bulk of the tumor, represented by epithelial cells, but also in the elimination of the staminal compartment of the tumor, which is responsible for breast cancer metastatization and potential resistance to chemotherapy. Moreover, being a transmembrane protein, TENM4 can be targeted by both the humoral and cellular responses that may be induced by anti-TENM4 vaccination. The safety of TENM4 immune-targeting, by means of anti-cancer vaccination, could be guaranteed by the fact that its low expression is restricted to the nervous system, which is an immune privileged site. Nevertheless, considering reports of adoptive cell therapy for melanoma which targeted a visceral metastasis, and which also cross-reacted with a central nervous system antigen, causing significant toxicity, the safety issues potentially related to anti-TENM4 vaccinations need to be deeply investigated in in vivo systems, as do the vaccine’s immunogenicity and efficacy, which could be limited by the host immune tolerance against the self-antigen.

The potential of immunotherapeutic strategies to overcome immune tolerance and induce an effective antitumor response could only be explored by means of valuable pre-clinical mouse models of breast cancer. In this panorama, few data on suitable models of TENM4-positive breast cancer are currently available. We have therefore demonstrated the potential relevance of 4T1, TUBO, and BALB-neuT tumors as appealing models with which to study the involvement of TENM4 in TNBC- and HER2-positive breast cancer’s initiation and progression, and the clinical impact of its immune-targeting.

Despite their limited relevance for immunological studies, the use of xenografts with human breast cancer cells, such as MDA-MB-231, for translational purpose may be of paramount importance for gaining insight into the role of TENM4 in the biology, progression, and metastatization of human TNBC. Moreover, the effects of TENM4 immunotargeting on human TNBC progression could also be elucidated by using adoptive transfer experiments in which anti-TENM4 antibodies and specific T cells, induced in immunocompetent mice, are injected into MDA-MD-231-tumor-bearing mice.

Considering the role of miR-708 in breast cancer progression and resistance to therapy, further investigations to evaluate the expression of this miRNA in both the murine and human preclinical mouse models of TNBC- and HER2-positive tumors could prove to be useful. Interestingly, MDA-MB-231 tumors grown in immunodeficient mice and orthotopic models have also been used to demonstrate the role of miR-708 downregulation in metastatization and the efficacy of its potential delivery for therapeutic purposes [77,85]. These data lay the foundation for the design of combinatorial treatments that consist of TENM4 immune targeting and miR-708 administration into the tumor. Even if no data on the expression of miR-708 and its potential role in 4T1, TUBO, and BALB-neuT tumors are currently available, they can be easily obtained and used to evaluate the possibility of testing combinatorial treatments also in immunocompetent preclinical models of TNBC- and HER2-positive breast cancer.

In conclusion, despite the relative lack of data on TENM4, it is emerging as an appealing shared oncogene in different breast cancer subtypes and deserves further investigations that will likely validate its clinical relevance as a marker and immunotherapeutic target for more effective treatment of this still-fatal disease. Moreover, the significant increase in the TENM4 mRNA level observed in colorectal tumors, compared to normal colon tissue in a mouse model of colorectal cancer [45] may suggests that TENM4 may become an interesting and suitable target, not only for breast cancer, but also for other tumor histotypes.

## Figures and Tables

**Table 1:** MiR-708 target genes in different tumor types. Red arrows represent tumors in which miR-708 or its targets are upregulated. Blue arrows represent tumors in which miR-708 or its targets are downregulated.

	TUMOR TYPE		TARGET GENES
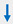	Prostate cancer	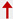	*KPNA4, CD44, AKT2, NNAT*
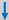	Gastric cancer	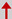	*Notch 1*
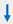	Hepatocellular carcinoma	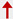	*SMAD3*
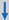	Renal cell carcinoma	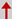	*ZEB2, Survivin, FLIP*
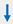	Ewing sarcoma	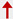	*EYA3*
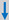	Ovarian cancer	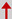	*Caspase-3*
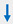	Melanoma	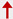	*LEF1*
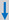	Osteosarcoma	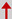	*URGCP*
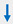	Chronic lymphocytic leukemia	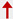	*IKKβ*
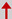	Lung cancer	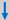	*p21*
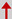	Colorectal cancr	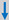	*CKN2B*
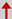	Bladder cancer	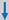	*Caspase-2*

**Table 2:** MiR-708 target genes in breast cancer. Red arrows represent upregulated MiR-708 targets. Blue arrows represent miR-708 downregulation in breast cancer.

	TUMOR TYPE		TARGET GENES
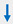	**Breast cancer**	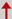	*Neuronatin*
		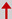	*LSD1*
		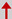	*ZEB1*
		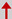	*CDH2*
		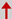	*Vimentin*
		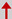	*CD47*
		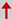	*IKKβ*

## Data Availability

Not applicable.

## Supplementary Materials

The following supporting information can be downloaded at: https://www.mdpi.com/article/10.3390/cells11010172/s1, Figure S1: Schematic drawing of the positions of TENM4 mutations across the aminoacidic sequence of the protein; Table S1: TENM4 mutation, deletion, alteration position code in breast cancer.
