# Supplementary material for: Role and Involvement of TENM4 and miR-708 in Breast Cancer Development and Therapy
Source: Cells. 2022 Jan 5;11(1):172. doi: 10.3390/cells11010172 (PMC8750459; doi:10.3390/cells11010172)
Supplement: Supplementary file 1 [file cells-11-00172-s001.zip › Supplementary Figure S1.pptx]

## Slide 1
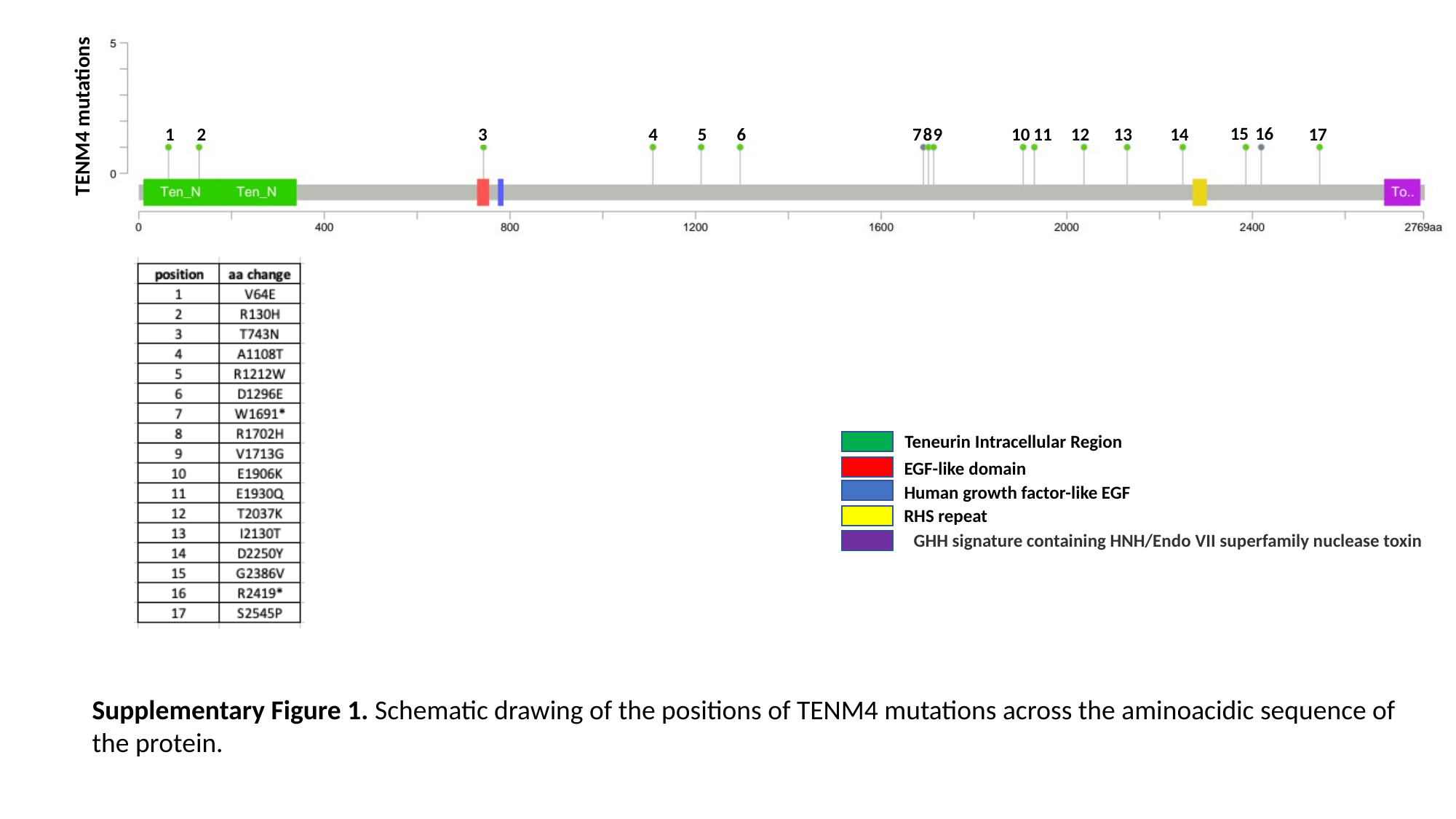

16
15
1
2
3
4
5
6
7
8
9
10
11
12
13
14
17
TENM4 mutations
Teneurin Intracellular Region
EGF-like domain
Human growth factor-like EGF
RHS repeat
GHH signature containing HNH/Endo VII superfamily nuclease toxin
Supplementary Figure 1. Schematic drawing of the positions of TENM4 mutations across the aminoacidic sequence of the protein.
